# Supplementary material for: Developmental single-cell transcriptomics of hypothalamic POMC neurons reveal the genetic trajectories of multiple neuropeptidergic phenotypes
Source: eLife. 2022 Jan 19;11:e72883. doi: 10.7554/eLife.72883 (PMC8806186; doi:10.7554/eLife.72883)
Supplement: Transparent reporting form [file elife-72883-transrepform1.docx]

***eLife’s* transparent reporting form**

We encourage authors to provide detailed information *within their submission* to facilitate the interpretation and replication of experiments. Authors can upload supporting documentation to indicate the use of appropriate reporting guidelines for health-related research (see [EQUATOR Network](http://www.equator-network.org/%20)), life science research (see the [BioSharing Information Resource](https://biosharing.org/)), or the [ARRIVE guidelines](http://www.plosbiology.org/article/info:doi/10.1371/journal.pbio.1000412) for reporting work involving animal research. Where applicable, authors should refer to any relevant reporting standards documents in this form.

If you have any questions, please consult our Journal Policies and/or contact us: [editorial@elifesciences.org](mailto:editorial@elifesciences.org).

**Sample-size estimation**

- You should state whether an appropriate sample size was computed when the study was being designed
- You should state the statistical method of sample size computation and any required assumptions
- If no explicit power analysis was used, you should describe how you decided what sample (replicate) size (number) to use

Please outline where this information can be found within the submission (e.g., sections or figure legends), or explain why this information doesn’t apply to your submission:

We combined at least 6 embryos/pups from 2 dams to isolate cells for single cell sequencing at each individual developmental stage. Pooled sample sizes (n>=6) was sufficient to provide a reasonable number of cells for single cell-type analysis. Both male and female embryos/pups were used for cell isolation, but the data were not analysed using sex as an independent variable because of the uncertainty of accurately assigning biological sex to each single cell.

The detailed information can be found in the Material and Methods section.

**Replicates**

- You should report how often each experiment was performed
- You should include a definition of biological versus technical replication
- The data obtained should be provided and sufficient information should be provided to indicate the number of independent biological and/or technical replicates
- If you encountered any outliers, you should describe how these were handled
- Criteria for exclusion/inclusion of data should be clearly stated
- High-throughput sequence data should be uploaded before submission, with a private link for reviewers provided (these are available from both GEO and ArrayExpress)

Please outline where this information can be found within the submission (e.g., sections or figure legends), or explain why this information doesn’t apply to your submission:

We pooled at least 6 embryos/pups from 2 dams to reduce inter animal variability. To validate the defined cell clusters based on *Pomc* counts >= 1 UMI, we performed an independent unsupervised cluster analysis of the transcriptomes from cell that had *DsRed* counts of >= 1 UMI. The results from the two analyses were consistent with each other. The RNAScope images were obtained from at least two independent embryonic brain sections. The TRAP-seq dataset was generated using three biological replicates. At least 5 animals from P12 or P60 were pooled together to generate each of the three biological replicates. This protocol was recommended to us by our local bioinformatics core linked to the Sequencing Core at UM. Data analysis was based on combining all three biological replicates.

We applied different criteria on each developmental stage to filter out possible doublets and low quality cells in the scRNAseq raw data. Specifically, at E11.5, we removed outlier cells that hadUMI counts over 60,000 or less than 5,000, and gene counts over 8,000 or less than 1,000 (determined by the visualization of UMI counts and gene counts distribution). At E13.5, we removed outlier cells that had UMI counts over 30,000 or less than 2,500, and gene counts over 1,500. At E15.5, we removed outlier cells that had UMI counts over 40,000, and gene counts over 6,000 or less than 1,000. At E17.5, we removed outlier cells that had UMI counts over 25,000, and gene counts over 5,000 or less than 1,000. At P5, we removed outlier cells that had UMI counts over 60,000, or less than 1,500 and gene counts over 8,000 or less than 1,500. At P12, we removed outlier cells that had UMI counts over 60,000, or less than 2,000 and gene counts over 8,000 or less than 1,500. Moreover, cells with a high proportion of mitochondrial genes (>10%) or a high proportion of hemoglobin genes (>10%) were filtered out. Finally, we removed all the cells without any *Pomc* transcript UMI counts. A total of 13,953 cells passed the exclusion criteria (E11.5: 1,498, E13.5: 1,796, E15.5: 2,078, E17.5: 1,139, P5: 3,909 and P12: 3,533) for downstream analysis.

The detailed information was presented in the Material and Methods section.

All raw sequencing data have been deposited in the Gene Expression Omnibus under accession numbers GSE154153 and GSE181539. GSE154153 is already publically available. The link for GSE181539 is https://www.ncbi.nlm.nih.gov/geo/query/acc.cgi?acc=GSE181539. Please log in with the reviewer token gbcvaiayhzuxpur.

The detailed information can be found in the Accession Codes part.

**Statistical reporting**

- Statistical analysis methods should be described and justified
- Raw data should be presented in figures whenever informative to do so (typically when N per group is less than 10)
- For each experiment, you should identify the statistical tests used, exact values of N, definitions of center, methods of multiple test correction, and dispersion and precision measures (e.g., mean, median, SD, SEM, confidence intervals; and, for the major substantive results, a measure of effect size (e.g., Pearson's r, Cohen's d)
- Report exact p-values wherever possible alongside the summary statistics and 95% confidence intervals. These should be reported for all key questions and not only when the p-value is less than 0.05.

Please outline where this information can be found within the submission (e.g., sections or figure legends), or explain why this information doesn’t apply to your submission:

The cell numbers from each developmental stage and each cluster were indicated in Figure 1.

The identification of differentially expressed genes in each cluster / group was conducted using Seurat package with the Wilcoxon Rank Sum test.

The exact average gene expression and p-values adjusted for multiple comparisons were reported in the Supplemental Tables.

The differentially enriched genes from TRAP-seq datasets were acquired using DEseq2 package with paired-samples and treatment (pull-down vs. supernatant) as the main effects. The raw counts were uploaded to GEO (accession #: GSE181539). The normalized reads (counts per million) and exact p-values were reported in Supplemental Tables 12 and 13.

(For large datasets, or papers with a very large number of statistical tests, you may upload a single table file with tests, Ns, etc., with reference to sections in the manuscript.)

**Group allocation**

- Indicate how samples were allocated into experimental groups (in the case of clinical studies, please specify allocation to treatment method); if randomization was used, please also state if restricted randomization was applied
- Indicate if masking was used during group allocation, data collection and/or data analysis

Please outline where this information can be found within the submission (e.g., sections or figure legends), or explain why this information doesn’t apply to your submission:

Random grouping is not applicable for this study since all single cells were collected from different groups according to their development stage.

Clustering of single cells was performed in an unbiased and blinded manner using Seurat package. Cluster names were assigned after generation of clusters.

The detailed information on the clusters can be found in the Results section.

**Additional data files (“source data”)**

- We encourage you to upload relevant additional data files, such as numerical data that are represented as a graph in a figure, or as a summary table
- Where provided, these should be in the most useful format, and they can be uploaded as “Source data” files linked to a main figure or table
- Include model definition files including the full list of parameters used
- Include code used for data analysis (e.g., R, MatLab)
- Avoid stating that data files are “available upon request”

Please indicate the figures or tables for which source data files have been provided:

No custom code was used in this study. The detailed analytical methods with related software packages were described in the methods. The numerical data used to create both main and supplementary figures were included in detailed excel-generated worksheets as annotated Supplemental Tables.
